# Supplementary material for: Attitude towards healthy nutrition and mental toughness: a study of taekwondo athletes
Source: PeerJ. 2024 Mar 29;12:e17174. doi: 10.7717/peerj.17174 (PMC10984172; doi:10.7717/peerj.17174)
Supplement: Supplemental Information 1 [file peerj-12-17174-s001.pdf]

STROBE Statement—Checklist of items that should be included in reports of ***cross-sectional studies***

	Item No	Recommendation	Page No
**Title and abstract**	1	(*a*) **Attitude Towards Healthy Nutrition and Mental Toughness: A Study of Taekwondo Athletes.** We aim to explore the potential relationship between taekwondo athletes' attitudes towards healthy nutrition and their mental toughness. This study employed a relational screening model, a research methodology designed to evaluate the presence and magnitude of change between two or more variables	1
		(*b*) Descriptive statistics, correlation analysis, and regression analysis were performed to explore the relationship between the variables within the research model. The correlation analysis identified two significant, positive, and moderate correlations: (1) between *Knowledge About Nutrition* and mental toughness ($r=0.626, p<0.001$) and (2) between *Positive Nutrition* and mental toughness ($r=0.672, p<0.001$). The regression analysis revealed that both *Knowledge About Nutrition* ($\beta=0.360, p<0.001$) and *Positive Nutrition* ($\beta=0.461, p<0.001$) significantly contribute to mental toughness. The findings suggest that as athletes' knowledge of nutrition expands and their attitudes towards healthy eating become more positive, their mental toughness also appears to improve.	1
**Introduction**			
Background/rationale	2	Taekwondo is not solely rooted in tradition. It has ascended to the world stage, securing its place as an Olympic sport, transforming into a contemporary sport with enduring appeal. With its deep cultural roots and emphasis on physical training, taekwondo offers a captivating blend of tradition and personal empowerment. Its accessibility lies in its simple and easily learnable movements, making it welcomed by diverse audiences. Across the world of sports, from athletes to coaches to researchers, mental toughness is hailed as a key ingredient for success. It serves as a crucial element contributing to athletic achievement. Enhancing individuals' nutritional knowledge and fostering positive attitudes towards healthy dietary practices have been identified as key drivers of overall well-being. On the other hand, malnutrition or nutritional deficiencies may contribute to negative emotional states. Therefore, fostering nutritional knowledge and implementing improved dietary practices not only enhances physical health but also safeguards mental well-being. In this context, proper nutrition plays a pivotal role in optimizing exercise performance, fueling our bodies with the energy needed to thrive during physical exertion. Maintaining both physical and mental well-being demands a harmonious union of healthy eating and regular exercise. As the cornerstone of optimal athletic performance, both physically and mentally, athletes face particular pressure to prioritize healthy dietary habits. Though research specifically examining the interplay between healthy eating and attitudes towards mental toughness remains limited, this study aims to contribute to a more comprehensive understanding of this crucial interrelationship.	2-3
Objectives	3	The specific focus of this investigation was on the potential relationship between attitude towards healthy nutrition and mental toughness in athletes. The hypotheses created within the scope of the research model are presented below.<br>H1: Knowledge about nutrition has positive effects on mental toughness.<br>H2: Emotion towards nutrition has positive effects on mental toughness.<br>H3: Positive nutrition has positive effects on mental toughness.<br>H4: Malnutrition has negative effects on mental toughness.	4
**Methods**			
Study design	4	Relational screening model was employed in this study.	4
Setting	5	During a three-month period (February 10 to May 25, 2022), researchers gathered data from licensed taekwondo athletes in Osmaniye, Turkey.	4

Participants	6	(*a*) The participants were recruited through a convenience sampling method, primarily among readily available taekwondo athletes in Osmaniye. These included individuals aged 10 and above attending training sessions at local clubs (Turkey) within the data collection timeframe. Importantly, all participants opted to participate in the study voluntarily.	4
Variables	7	Outcome: Mental toughness (dependent variable measured using a validated instrument).<br>Exposure: Attitude towards healthy eating (independent variable measured using a validated scale).<br>Potential confounders: Physical activity level, sleep quality, and injury history.<br>Effect modifiers: gender and competition level (the relationship may be stronger for elite athletes compared to recreational athletes).	4
Data sources/ measurement	8*	Data collection utilized a comprehensive questionnaire with three distinct sections. Part 1 gathered demographic information (gender, age) to provide context for the analysis. Part 2 delved into the athletes' attitudes towards healthy eating habits, exploring their preferences and beliefs. Finally, Part 3 focused on assessing their mental toughness, measuring their resilience and perseverance.	4
Bias	9	To prioritize data quality and accuracy, the study focused on objective measures and standardized procedures rather than relying solely on subjective responses.	5
Study size	10	Employing a voluntary participation model, the study facilitated the involvement of all athletes expressing interest, thereby optimizing the sample size and Promoting inclusivity. Due to logistical limitations, the study participants were drawn from the two official taekwondo halls within the research city, offering a focused sample within a specific training context.	4
Quantitative variables	11	Through correlation and regression analyses, we aimed to uncover the patterns and dependencies, if any, existing between the chosen variables, quantifying their association strength and direction.	5
Statistical methods	12	(*a*) The study employed various statistical techniques to assess the validity and reliability of the scales used and to investigate the hypothesized relationships between them.<br>Confirmatory Factor Analysis (CFA): CFA was conducted to examine the factor structure of each scale, evaluating whether the measured items adequately reflected the underlying theoretical constructs.<br>Internal Consistency: Cronbach's alpha coefficients were calculated to assess the internal consistency of each scale.<br>Pearson Correlation Analysis: This bivariate analysis was used to examine the linear relationships between the two constructs of interest, namely attitude towards healthy nutrition and mental toughness.<br>Multiple Regression Analysis: This multivariate technique was employed to further investigate the influence of attitude towards healthy nutrition on mental toughness, while controlling for any other relevant variables.	5
		(*b*) There is only one group (taekwondo athletes) in the study.	4
		(*c*) Data cleaning procedures ensured a complete dataset with no missing information for any participants or variables.	5
		(*d*) Convenience sampling method was utilized.	5
		(*e*) Descriptive Statistics: Descriptive statistics, such as means, standard deviations, and frequency distributions, were computed for all variables to provide a basic understanding of the data.	5
**Results**			
Participants	13*	(a) Athletes aged under 10 years old were excluded from the study, as their developmental stage might limit their ability to fully understand and respond to the research questions.	4
		(b) Participation in the study was voluntary, and only those who consented were included in the analysis. This reflects the focus on ethical research practices and individual choice.	4

Descriptive data	14*	(a) The study group comprised 276 active and licensed taekwondo athletes who voluntarily participated in the study (*Age M=17.18 ± SD= 7.13, N=125 women, N=151 men*).	4
		(b) The meticulous research design resulted in a pristine dataset free from missing data, bolstering the confidence in the findings.	5
Outcome data	15*	The study recruited a diverse group of 276 licensed taekwondo athletes, with a near- balanced representation of females (45.3%) and males (54.7%).	4
Main results	16	(*a*) To evaluate the model's explanatory power, both the R^2 and adjusted R^2 were calculated and presented in the analysis. The R^2 reflects the overall variance explained by the model, while the adjusted R^2 accounts for the model's complexity.	6
		(*b*) The study's sample included taekwondo athletes representing both genders female and male.	4
		(*c*) Since the study design focused on exploring the relationship between variables rather than comparing risk factors, it does not provide an estimate of relative risk.	5
Other analyses	17	The data analysis strategy relied on a combination of descriptive statistics, correlation and regression methods, along with reliability analysis to ensure consistency of the measurements. No additional analytical approaches were used.	5
**Discussion**			
Key results	18	As athletes' understanding of nutrition and their appreciation for healthy eating habits grew, their mental toughness also seemed to strengthen, suggesting a synergetic relationship between nutritional knowledge, positive attitudes, and mental toughness.	7
Limitations	19	Generalizability: The study results are limited to taekwondo athletes and may not be generalizable to other athlete populations or the general public. Factors specific to taekwondo culture or training might influence the relationship between nutrition knowledge, attitudes, and mental toughness, making it difficult to extrapolate the findings to other contexts. Selection bias: Although no intentional bias was mentioned, the convenience sampling method used to recruit participants could potentially introduce selection bias. The athletes who chose to participate might be more interested in nutrition or have different characteristics than the broader taekwondo population. Confounding variables: The study design might not account for potential confounding variables that could influence the observed relationship between variables. For example, physical activity level, injury history, or socioeconomic status could influence both nutrition knowledge and mental toughness, making it difficult to isolate the specific effect of one on the other. Temporal limitations: The study was conducted within a specific timeframe and may not reflect changes in taekwondo training, athlete demographics, or nutritional trends over time. Cultural limitations: The research was conducted in Osmaniye, Turkey, and the cultural context might influence factors like attitudes towards healthy eating and mental toughness. Applying the findings to athletes from different cultural backgrounds might require caution.	9
Generalisability	21	The current study's findings, while insightful for taekwondo athletes, have limitations in generalizability beyond this specific population. The convenience sampling method and focus on a single sport limit the ability to extrapolate the results to other athletes or the general public. Factors specific to taekwondo culture, training demands, and demographics might influence the observed relationships between nutrition knowledge, attitudes, and mental toughness. However, considering the growing body of research exploring the mental aspects of athletic performance, this study contributes valuable groundwork for future investigations. Replication and extension of these findings with larger, more diverse samples across different sports could solidify the potential link between nutrition and mental resilience in athletes. Such research, involving collaboration with large research groups and athletes from various disciplines, will be crucial for establishing broader generalizability and translating these insights into practical interventions for athlete development.	9

**Other information**			
Funding	22	This research was conducted without financial support from any external sources, including grants, sponsorships, or industry collaborations. The researchers independently financed all aspects of this study.	10

*Give information separately for exposed and unexposed groups.

Note: An Explanation and Elaboration article discusses each checklist item and gives methodological background and published examples of transparent reporting. The STROBE checklist is best used in conjunction with this article (freely available on the Web sites of PLoS Medicine at http://www.plosmedicine.org/, Annals of Internal Medicine at http://www.annals.org/, and Epidemiology at http://www.epidem.com/). Information on the STROBE Initiative is available at www.strobe-statement.org.
